# A 4E (energy, exergy, environmental, and economic) evaluation of a solar air heater with airflow beneath a V-shaped perforated finned absorber

**DOI:** 10.1038/s41598-026-41661-6

**Published:** 2026-05-18

**Authors:** Fatma M. Shaaban, Mahmoud A. Abdelhamid, Moustafa H. Abozid, Shaimaa A. Hassan, Mohamed F. Abouelenein, Mohamed F. Atia

**Affiliations:** 1 Department of Agricultural Engineering, Faculty of Agriculture, Ain Shams University, Cairo, 11241 Egypt; 2 College of Business, Imam Mohammad Ibn Saud Islamic University (IMSIU), Riyadh, 11432 Saudi Arabia

**Keywords:** 4E analysis, CO_2_ mitigation, V-shaped fins, solar air heater configurations, sustainable, thermal efficiency, Energy science and technology, Engineering, Environmental sciences

## Abstract

Egypt has high levels of solar radiation throughout the year, making solar air heaters (SAHs) a practical and economical way to support sustainable energy applications and reduce reliance on traditional energy sources. This study experimentally investigates the energy, exergy, environmental, and economic (4E) performance of three SAH configurations under different air mass flow rates. The experiment was conducted at the Faculty of Agriculture, Ain Shams University, Egypt (30°11′ N, 31°24′ E). The tested configurations include an airflow channel above the absorber plate (SAH1), an airflow channel below the absorber plate (SAH2), and a modified design with airflow below the absorber plate integrated with perforated V-shaped fins (SAH3). The experiments were conducted during July 2025, and real-time monitoring of temperatures, solar radiation, and airflow was carried out. At a mass flow rate of 0.009 kg/s, SAH3 achieved the highest average thermal efficiency of 62%, compared with 53% for SAH2 and 44% for SAH1. Regarding exergy efficiency at 0.006 kg/s, SAH3 achieved an average of 3.7%, exceeding those of SAH2 (2.6%) and SAH1 (1.8%). At 0.009 kg/s, the SAH3 achieved the lowest energy cost of 0.0003 $/kWh and mitigated CO_2_ emissions by approximately 1.07 tons annually, resulting in an estimated carbon credit of 53.43 USD. These results confirm that the proposed design is a promising option for sustainable solar thermal applications.

## Introduction

The development of renewable energy and clean technologies is essential for reducing dependence on conventional fossil fuels, promoting sustainability, minimizing carbon emissions, and mitigating environmental pollution. Technological innovations play a vital role in advancing energy conservation and supporting a just and green energy transition^1–4^. Egypt, with its high solar irradiance and extended sunshine duration, presents ideal conditions for solar energy applications^5^. In particular, SAHs offer a promising solution for harnessing solar energy to provide thermal energy for buildings, either through passive or active systems. SAHs are renewable energy systems that convert solar radiation into useful thermal energy for many applications, including space heating, agricultural drying, cooking, and industrial processes. Their simple design, ease of fabrication using locally available materials, and cost-effectiveness make them attractive for both rural and urban settings. However, their broader adoption is often limited by challenges such as low thermal efficiency and significant heat losses^6–8^. Therefore, increasing the heat transfer surface area using rough or structured surfaces in SAHs enhances turbulence within the air duct, which improves the convection heat transfer rate^9–12^.

Gao^13^ compared the thermal performance of three SAH configurations: two with cross-corrugated absorber plates and one with a flat plate. The reported thermal efficiencies were 58.9% and 60.3% for the cross-corrugated designs, and 48.6% for the flat plate. These results demonstrate that the cross-corrugated configurations significantly outperform the flat plate due to the increased turbulence and heat transfer rates induced by the surface geometry. The heat transfer enhancement in the corrugated channels was found to be approximately 3.25 times greater than that in the flat plate design. Maurya et al.^14^ conducted both experimental and numerical investigations on the thermo-hydraulic performance of tubular three-pass SAHs. The highest thermal efficiencies were observed at mass flow rates of 0.006, 0.004, and 0.002 kg/s, yielding efficiencies of 60.04%, 41%, and 33.3%, respectively. The study also revealed that increasing energy production through higher mass flow rates effectively reduces the energy cost per kWh, which ranged from $0.017 to $0.0311, depending on the airflow rate. Yeh et al.^15^ developed and analyzed a double-flow SAH through both theoretical and experimental approaches, concluding that the optimal air volume ratio between the upper and lower sub-channels was approximately 0.5. Kumar et al.^16^ analyzed a SAH with a circular finned absorber plate, varying fin heights from 5 mm to 25 mm and fins from 10 to 25. The optimal configuration for maximum heat transfer is found to be 15 fins. Karwa^17^ designed a SAH with an inclined finned absorber plate in equilateral triangular flow passages, demonstrating a thermal efficiency of 7.3–25.8% better than conventional designs due to increased effective heat transfer surface area. Alomar et al.^18^ modified a V-corrugated absorber plate (Model-1) and compared it with a normal V-corrugated collector (Model-2). The results indicate that Model-1 exhibits significantly higher thermal efficiency at 0.037 kg/s, with peak performance reaching 82.3% compared to 69.1% for Model-2. Therefore, SAH designs with modified absorber plates, whether corrugated, roughened, or finned, improve heat transfer, increase surface area, and achieve higher thermal efficiency.

In recent years, the environmental and economic performance of SAHs has gained increasing attention, as these systems not only contribute to reducing greenhouse gas emissions but also offer significant cost savings over their operational lifetime. Comprehensive evaluations that integrate aspects of energy, exergy, environmental, and economic (4E) provide a holistic understanding of SAH performance and sustainability potential. El-Bialy and Shalaby^19^ indicated that the finned plate SAH exhibits lower thermal efficiency compared to the V-corrugated plate design. The weaving-shaped plate SAH was found to have the highest cost per unit of energy at 0.0834 $/kWh, whereas the curved air jet impingement absorber plate SAH achieved the lowest cost per unit energy at 0.00133 $/kWh. Sharma and Dutta^20^ designed a SAH with an absorber plate fabricated from waste aluminum cans, achieving thermal efficiencies ranging from 52.58% to 63.65% and exergy efficiencies between 1.41% and 4.51%. Their work highlighted the energy, exergy, economic, and environmental benefits of this design in tea withering applications. Abo-Elfadl et al.^21^ assessed a double-pass SAH equipped with a pin-finned absorber, reporting higher energy and exergy efficiencies compared to flat absorbers, along with improved economic viability and environmental performance, particularly under the 2/3 double-pass airflow pattern. Arunkumar et al.^22^ demonstrated that an absorber plate configuration with multiple inlets achieved optimal thermo-hydraulic efficiencies of 84.5%, with an average enviro-economic value of 16 USD/year. Hegde et al.^23^ numerically examined a cross-flow SAH, reporting a maximum exergy efficiency of 5.28%, an environmental cost of 16.31 USD/year, and a CO_2_ mitigation rate of 1.12 t CO_2_/year. Ganesh Kumar et al.^24^ evaluated a SAH with a V-corrugated absorber plate, achieving a 15% increase in energy efficiency and a 34% improvement in exergy efficiency over conventional designs, while reducing CO_2_ emissions and enhancing economic and environmental performance by up to 23.4%. Abuşka^25^ evaluated V-groove and single-pass finned SAHs based on energy, exergy, and enviro-economic performance. The study reported exergy efficiencies ranging from 6% to 12% and thermal efficiencies between 43% and 60%. The corresponding enviro-economic cost values were estimated to range from $4.50 to $5.77 per year.

As mentioned above, numerous studies have investigated finned, corrugated, or rough-surfaced absorber plate designs to improve heat transfer in SAHs; however, most of these studies have focused on airflow above the absorber plate or solely on thermal performance. Accordingly, this study focuses on investigating airflow beneath the absorber plate integrated with V-shaped perforated fins and presents a comprehensive performance assessment from the perspectives of energy, exergy, environmental, and economic (4E) criteria. Three SAH configurations are experimentally examined under different air mass flow rates to evaluate their thermal and exergetic behavior. In addition, a detailed 4E analysis is conducted to identify the most efficient and economically viable configuration, thereby providing practical insights for improving the overall performance of solar air heating systems.

## Materials and methods

**Fig. 1:**
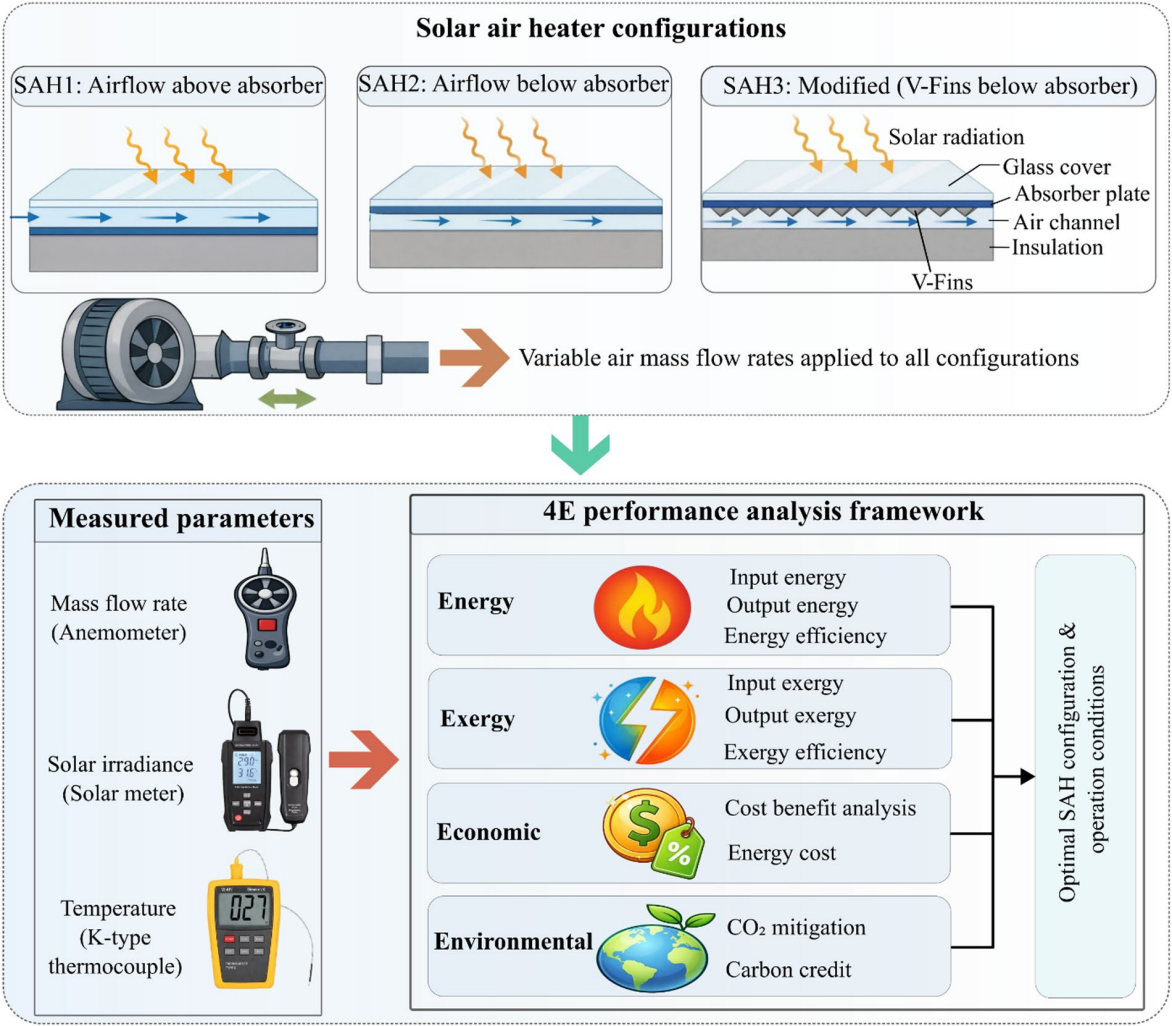
Schematic diagram of the experimental setup and methodology.

Figure 1 presents a schematic diagram of the experimental methodology used in this study. Three SAH configurations were tested under different mass flow rates. Solar irradiance, air flow rate, ambient air temperature, and inlet and outlet temperatures of the SAHs were measured. The data were collected to evaluate the 4E criteria.

### Configuration and specifications of the tested SAHs

**Fig. 2:**
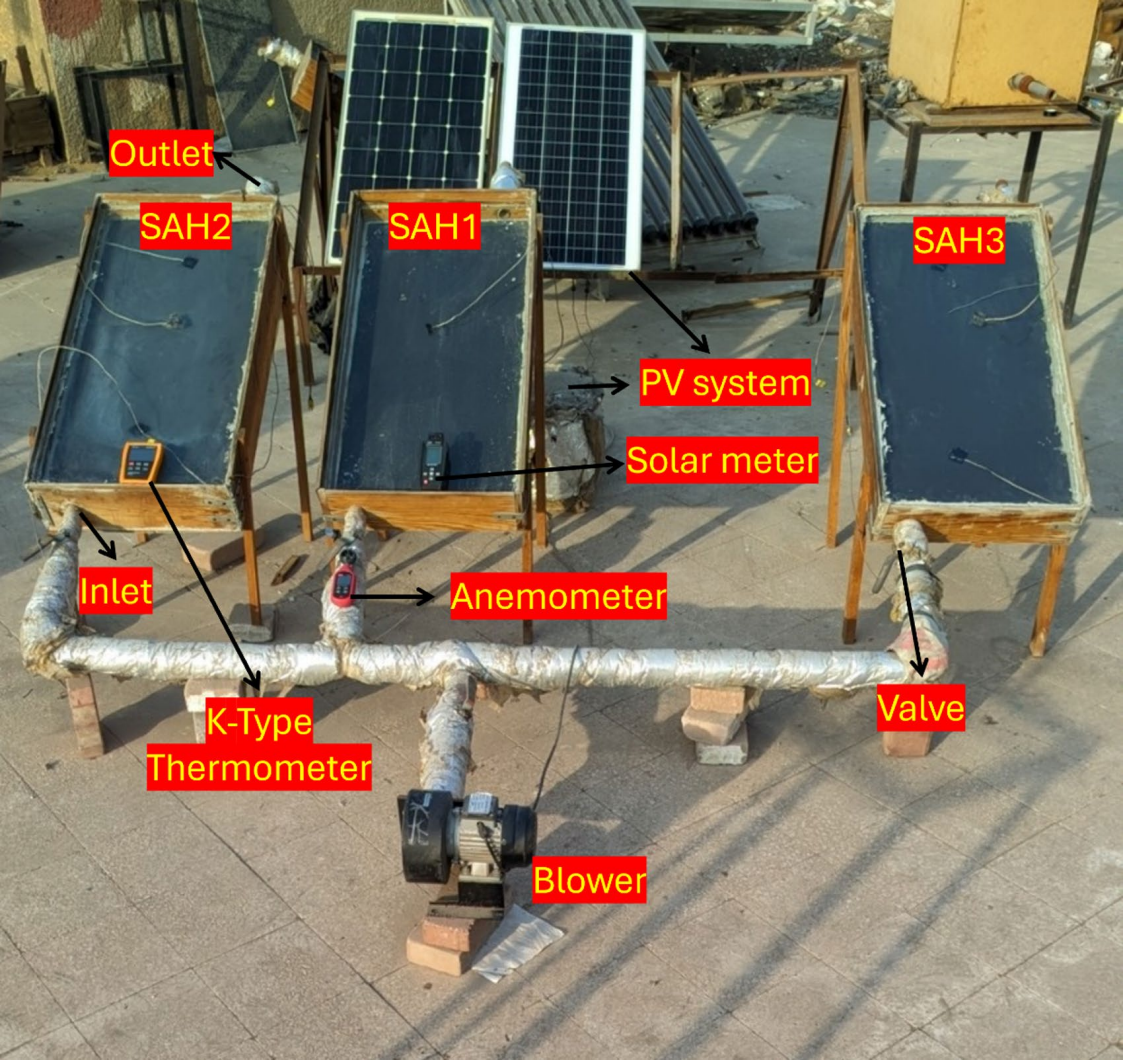
Photograph of experimental setup.

**Fig. 3:**
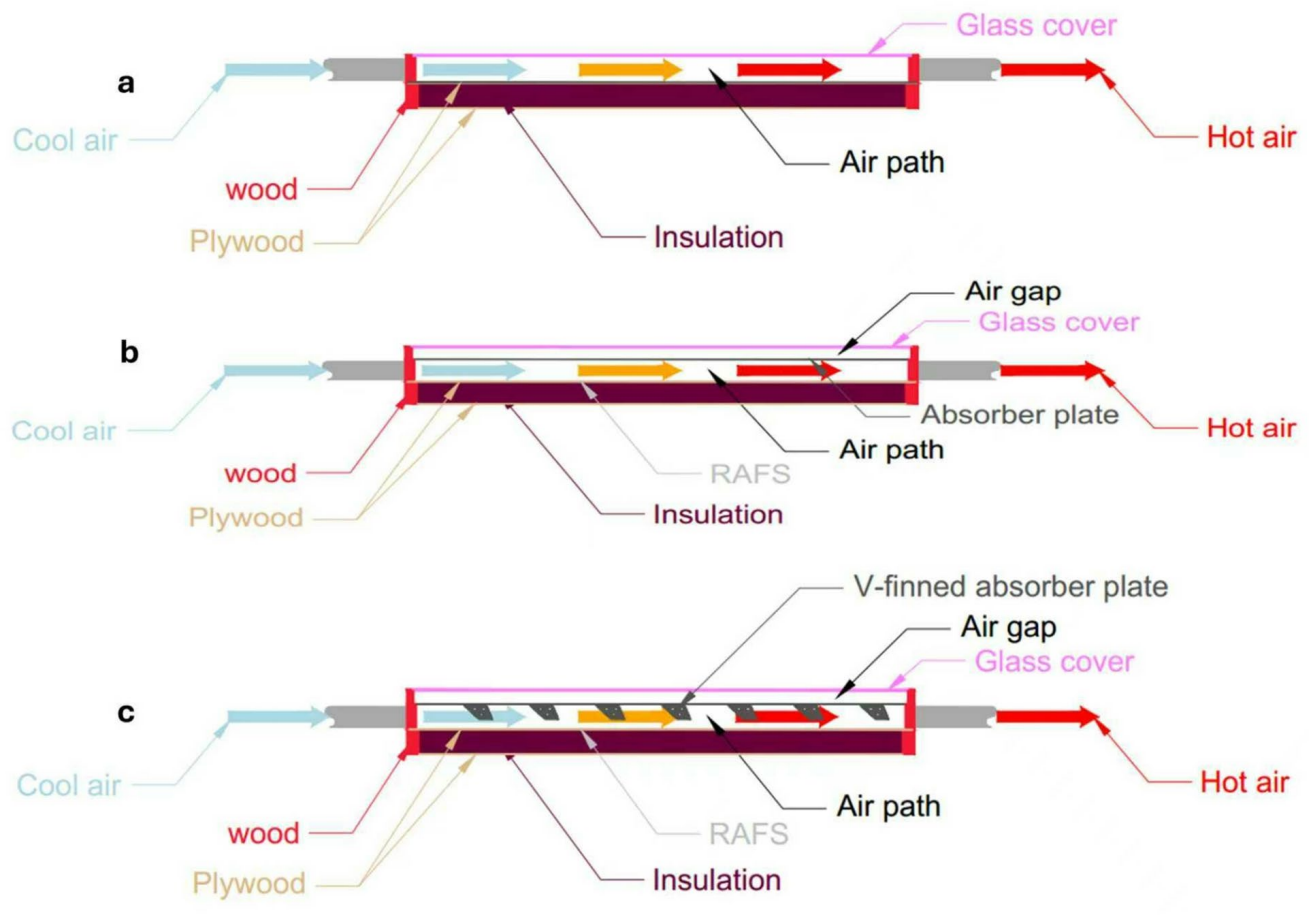
A sectional view of SAH1 (**a**), SAH2 (**b**), and SAH3 (**c**).

An experimental setup was carried out at the Faculty of Agriculture, Ain Shams University, Egypt (30°11′ N, 31°24′ E), to evaluate the performance of three different SAH systems. A photograph of the experimental setup is displayed in Fig. 2. As illustrated in Fig. 3, three different SAH configurations are used: (i) SAH1 design featuring an air chamber above the absorber plate (Fig. 3a), (ii) SAH2 design incorporating an air chamber beneath the absorber plate (Fig. 3b), and (iii) SAH3 design incorporating an air chamber beneath the absorber plate integrated with perforated V-shaped fins (Fig. 3c). The components of each SAH are a glass cover, absorber plate, insulation, and supporting frame. The SAH box, constructed from wood, measures 1 m × 0.5 m × 0.15 m. For SAH1, a 50 mm gap is maintained between the absorber plate and the glass cover, while a 30 mm spacing separates the absorber plate from the base of SAH2 and SAH3. The glass cover is sized 1 m × 0.5 m × 4 mm. To enhance corrosion resistance and solar absorption, the absorber plate was coated with matte black paint. Thermal insulation, 20 mm thick, was installed at the back of the collector to minimize heat loss. Airflow through the system was provided by an electric blower powered by a photovoltaic (PV) system. The detailed components of the SAH are listed in Table 1.

**Table 1:** Specifications of components, dimensions, and materials used in the SAH.

Component	Details
SAH box	1000 × 500 × 150 mm (internal); wood structure coated with black paint.
Inlet and outlet ducts	*Φ*40 mm; made of steel.
Glass cover	1000 × 500 mm, thickness 4 mm; glass with transmissivity of 0.9 and emissivity of 0.85.
Absorber plate	1000 × 500 mm, thickness 1 mm; aluminium with emissivity 0.9, absorptivity 0.9, and black paint coating.
RAFS	Reflective aluminum foil sheet
Insulation	Glass wool of thickness 50 mm

Using a PV-powered fan eliminates dependence on the electrical grid, reduces operational carbon emissions, and ensures sustainable, off-grid operation of the SAHs, making the system more environmentally friendly and economically viable over its lifespan. The PV system consisted of a 200 W PV panel, a charge controller, an inverter, and a battery. The PV system was properly sized to match the operational requirements of the SAH’s blower. The blower has a rated power of 180 W (220 V AC) and operates for 5 h daily (11:00 AM–4:00 PM), resulting in a daily energy demand of approximately 900 Wh. Considering inverter losses (≈ approximately 85%), the required battery-side energy is approximately 1,060 Wh/day. A standalone 12 V system was adopted, and a 120 Ah deep-cycle gel battery was selected, providing sufficient capacity for one day of autonomy with a maximum depth of discharge of 80%.

The PV generation system consists of a 200 Wp monocrystalline panel controlled by a 20 A MPPT charge controller. Based on the local solar conditions in Cairo (average 5.5 peak sun hours/day), the selected panel adequately supplies the blower during the entire operating period, which coincides with peak solar hours, while simultaneously charging the battery. The battery’s capacity margin compensates for any minor energy shortfall. This configuration ensures reliable off-grid operation of the SAH during the experimental period without reliance on the electrical grid. The detailed specifications of the PV system components are listed in Table 2.

**Table 2:** Specifications of components and sizing of the PV system.

Component	Details
PV panel	200 Wp Monocrystalline
Battery	12 V, 120 Ah Deep Cycle Gel, stores energy for continuous operation.
Charge controller	20 A MPPT, regulates charging and protects the battery.
Inverter	500 W Pure Sine Wave, converts 12 V DC to 220 V AC for the blower.
Blower	2800 rpm, 180 W, voltage 220 V, flow rate 405 m^3^/hr, pressure 450 Pa, model: DF-2, China.

**Fig. 4:**
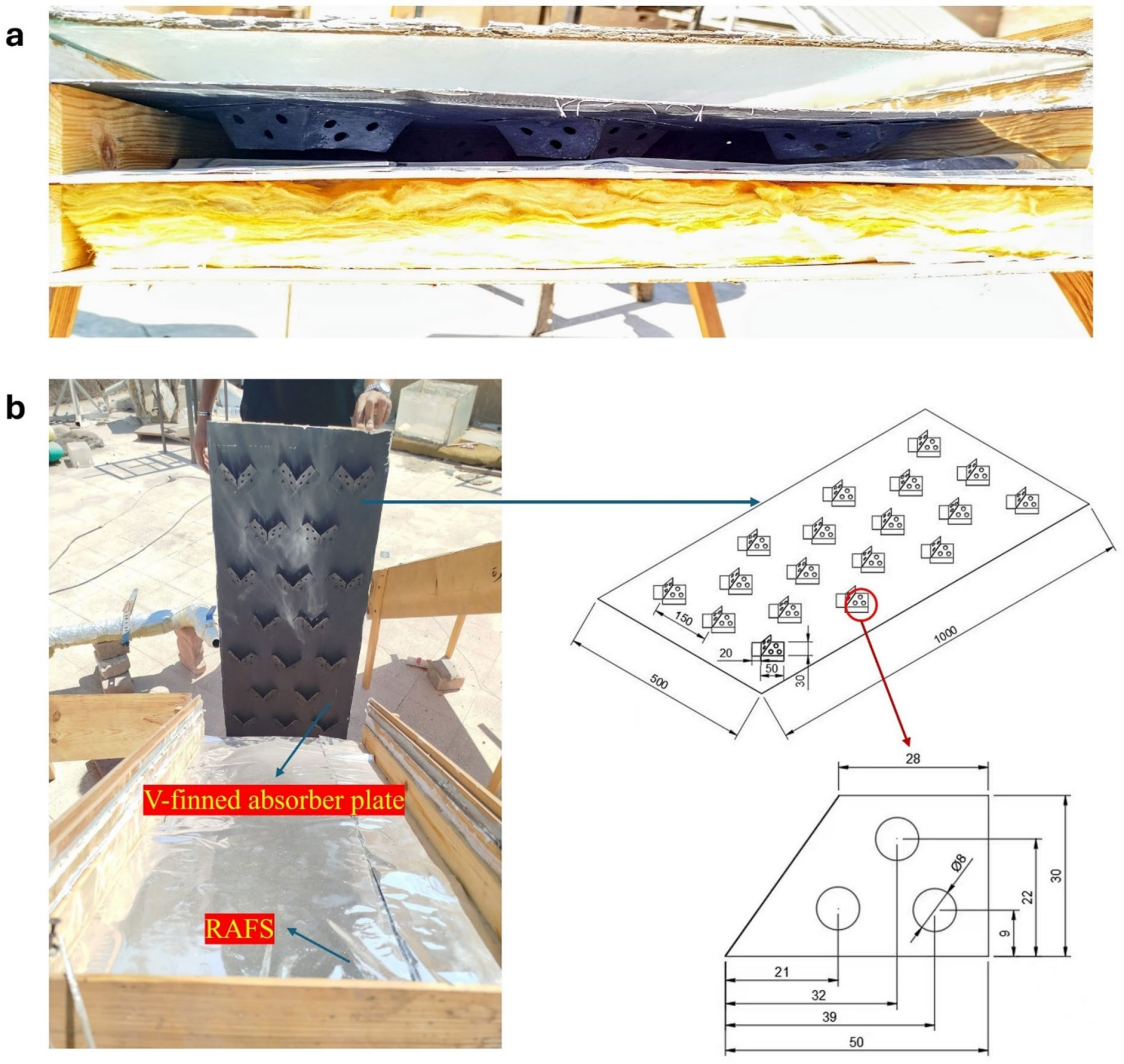
(**a**) Section view of the SAH3 and (**b**) image and schematic layout of the finned absorber plate.

Figure 4a presents a sectional view of the SAH3 configuration. The absorber plate, depicted in Fig. 4b, was fabricated from aluminum with dimensions of 1000 × 500 × 1 mm. To enhance heat transfer and improve the thermal performance of the system, specially designed perforated V-shaped fins were integrated into the absorber surface. These fins, made from aluminum with a thickness of 0.1 mm, were securely fixed and uniformly distributed across the absorber plate, as illustrated in Fig. 4b. The primary objective of incorporating these fins was to increase the surface area available for heat transfer, improve turbulence within the air channel, and thus enhance convective heat exchange between the absorber and the airflow.

### Measured parameters

To evaluate the performance of the SAHs, various parameters were measured during experiments conducted from 11:00 AM to 4:00 PM with an interval of 15 min. These parameters included the ambient air temperature (T_amb_), the air temperature at the inlet (T_a, i_), the air temperature at the outlet (T_a, o_), the mass flow rate of air ($\:{\dot{\mathrm{m}}}$), and the solar radiation intensity (I). The SAH systems were tested using different air mass flow rates: 0.006, 0.007, and 0.009 kg/s. The airflow rate was controlled using adjustable valves installed in the air duct. For each valve setting, the inlet air velocity was measured using a calibrated anemometer (UT-363, China) with an accuracy of ± 5% and was used to calculate the corresponding mass flow rate. The solar radiation was measured using a solar meter (CEM DT-1317, China) with an accuracy of ± 5%. Temperature measurements were carried out using a sheathed K-type thermocouple with an accuracy of ± 1 C, which was calibrated against a standard mercury-in-glass thermometer to ensure measurement accuracy. The work experiments are accomplished in the summer season in July 2025.

### Theory and analysis

**Fig. 5:**
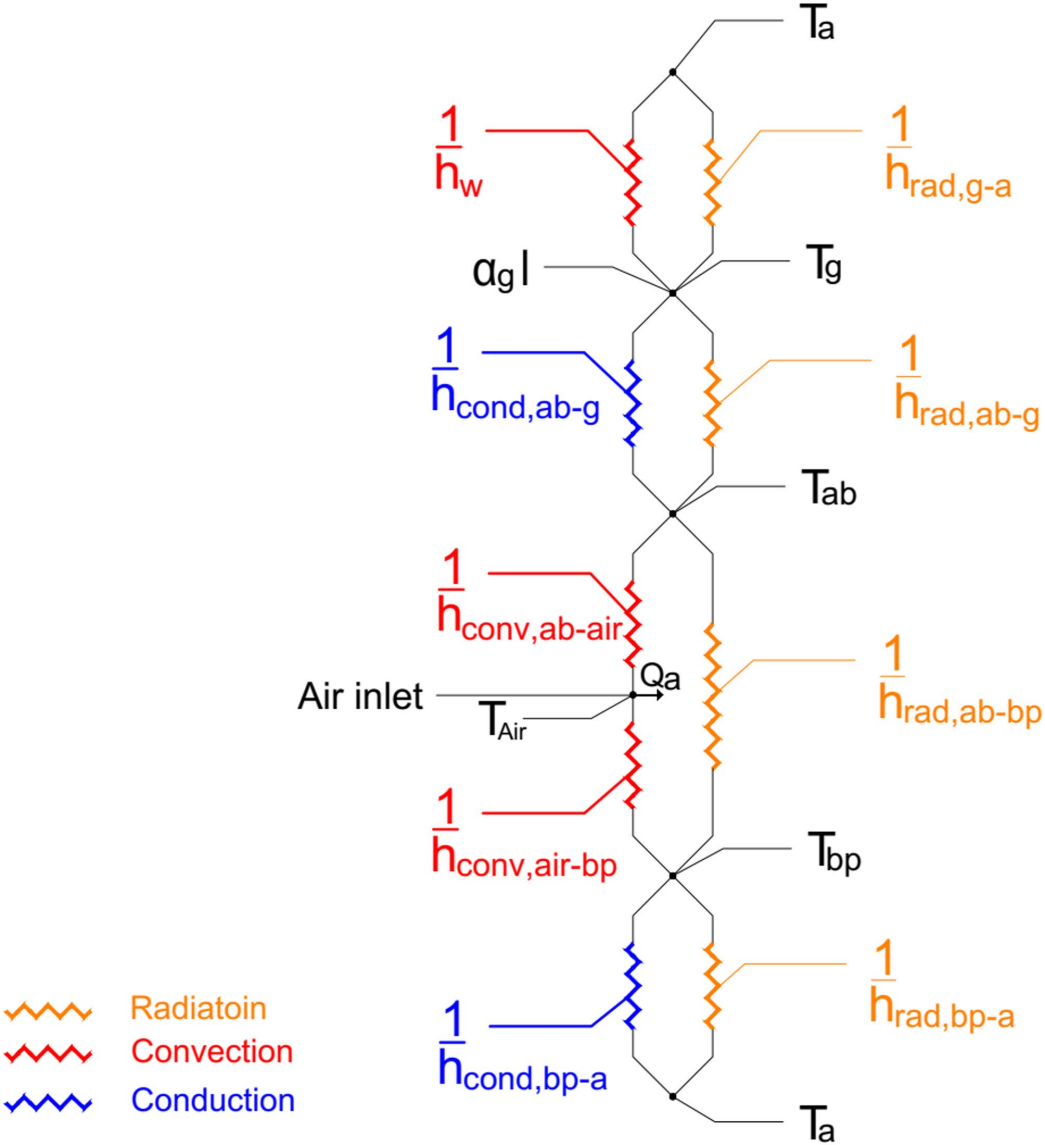
Thermal network for SAH3.

For modelling purposes, a number of simplifying assumptions are made to lay the foundations without obscuring the basic physical situation. These assumptions are:

1. The thermal performance of SAH is a steady-state.
2. Dust and dirt on the SAH and the shading of the heater absorbing plate are negligible.
3. Temperature of the air varies only in the flow direction.
4. Thermal losses through the heater backs are mainly due to the conduction across the insulation; those caused by the wind and the thermal radiation of the insulation are assumed to be negligible. The thermal network for the SAH3 is illustrated in Fig. 5.

The steady-state energy balance equations have been developed for different sections of the SAH.

The energy balance equation for the glass cover is expressed as ^26^:

1 $$\:{\alpha\:}_{g}I+{h}_{r,\:ab-g}\left({T}_{ab}-{T}_{g}\right)+{h}_{con,\:ab-g}\left({T}_{ab}-{T}_{g}\right)={h}_{W}\left({T}_{g}-{T}_{a}\right)+{h}_{r,g-sky}\left({T}_{g}-{T}_{sky}\right)$$

The radiative heat transfer is calculated as follows^27,28^:

2 $$\:{h}_{r,g-sky}=\sigma\:{\epsilon\:}_{g}\frac{({T}_{g}^{2}-{T}_{sky}^{2})({T}_{g}^{2}+{T}_{sky}^{2})}{({T}_{g}+{T}_{a})}$$

3 $$\:{h}_{r,ab-g}=\frac{\sigma\:({T}_{ab}^{2}+{T}_{g}^{2})({T}_{ab}+{T}_{g})}{\left(\frac{1}{{\epsilon\:}_{ab}}\right)+\left(\frac{1}{{\epsilon\:}_{g}}\right)-1}$$

The sky temperature *T*_*sky*_ is approximated as^29^:

4 $$\:{T}_{sky}=0.0552{{T}_{a}}^{1.5}$$

The convection heat transfer coefficient at the top surface is determined as^30^:

5 $$\:{h}_{w}=5.7+3.8\:{V}_{w}$$

The conductive heat transfer from the absorber plate to the glass cover is as follows:

6 $$\:{h}_{con,\:ab-g}=\frac{{k}_{air}}{L}\:\left({T}_{ab}-{T}_{g}\right)$$

The energy balance at the absorber plate is expressed as:

7 $$\:I\:{\tau\:}_{g}{\alpha\:}_{ab}\:={h}_{r,ab-bp}\left({T}_{ab}-{T}_{bp}\right)+{h}_{c,\:\:ab-air}\left({T}_{ab}-{T}_{air}\right)+{h}_{r,\:ab-g}\left({T}_{ab}-{T}_{g}\right)+{h}_{con,\:ab-g}\left({T}_{ab}-{T}_{g}\right)$$

The radiative heat transfer coefficient between the absorber and the baseplate is obtained as follows:

8 $$\:{h}_{r,ab-bp}=\frac{\sigma\:({T}_{ab}^{2}+{T}_{bp}^{2})({T}_{ab}+{T}_{bp})}{\left(\frac{1}{{\epsilon\:}_{ab}}\right)+\left(\frac{1}{{\epsilon\:}_{bp}}\right)-1}$$

The convective heat-transfer coefficient between the baseplate and the absorbing plate is calculated as:

9 $$\:{h}_{c,\:ab-bp}={Nu}_{\:ab-bp}\frac{{k}_{air}}{{d}_{f}}$$

The Nusselt number for natural convection in the channel below the absorbing plate ($\:{Nu}_{\:ab-bp}$) is obtained as^31^:

10 $$\:{Nu}_{ab-bp}=0.1673{\left({R}_{a}*\mathrm{cos}\theta\:\right)}^{0.2917}$$

The Rayleigh number (*R*_*a*_), defined as^31,32^:

11 $$\:{R}_{a}=\frac{{\rho\:}^{2}*{C}_{p}*g*\beta\:\left({T}_{ap}-{T}_{bp}\right){d}_{f}^{3}}{{k}_{air}*\mu\:}$$

The energy balance for the airflow in the channel:

12 $$\:{\dot{\mathrm{m}}}{C}_{p}\frac{\varDelta\:T}{\varDelta\:x}={h}_{c,\:ab-air}\left({T}_{ab}-{T}_{air}\right)-{h}_{c,\:air-bp}\left({T}_{air}-{T}_{bp}\right)$$

The convective heat transfer coefficient from the absorber plate to the air is obtained as follows^25^:

13 $$\:{h}_{c,\:ab-air}={Nu}_{\:ab-air}\frac{{k}_{air}}{{D}_{h}}$$

The Nusselt number for the absorber-air flow is calculated as^32^:

14 $$\:{Nu}_{\:ab-air}=0.0743{{R}_{e}}^{0.763}$$

The Reynolds number ($\:{R}_{e}$) is defined as^32^:

15 $$\:{R}_{e}=\frac{\rho\:*{V}_{air}*{D}_{h}}{\mu\:}$$

The hydraulic diameter $\:{D}_{h}$ is calculated as^25^:

16 $$\:{D}_{h}=\frac{4wh}{2w+2h}$$

The energy balance at the baseplate

17 $$\:{h}_{r,ab-bp}\left({T}_{ab}-{T}_{bp}\right)+{h}_{c,\:air-bp}\left({T}_{air}-{T}_{bp}\right)=\:{h}_{bp}\left({T}_{bp}-{T}_{a}\right)+{h}_{r,bp-a}\left({T}_{bp}-{T}_{a}\right)$$

The conduction heat-transfer coefficient ($\:{h}_{bp}$) across the insulation is estimated as:

18 $$\:{h}_{bp}=\frac{{k}_{ins}}{b}$$

### Performance parameters

#### Energy

The thermal efficiency of the SAHs is calculated using Eq. (19)^33,34^:

19 $$\:{\eta\:}_{th}=\frac{{Q}_{u}}{{IA}_{SAH}}\:\:\:\:\:\:\:$$

The useful heat gain ($\:{Q}_{u}$) is calculated using Eq. (20):

20 $$\:{Q}_{u}={\dot{\mathrm{m}}}_{a}\times\:{C}_{p,a}\times\:\left({T}_{a,o}-{T}_{a,i}\right)\:$$

To evaluate the effective efficiency (*η*_*eff*_), which accounts for the electrical energy consumed by the suction fan, Eq. (21) is used as:

21 $$\:{\eta\:}_{eff}=\frac{{Q}_{u}-{Q}_{fan}/CF}{{IA}_{SAH}}\:\:$$

where *CF* is the conversion factor, which converts electrical energy into its thermal energy equivalent. $\:CF$ value of 0.2 is typically assumed based on Debnath et al.^35^.

The pumping power *Q*_*fan*_ is calculated using Eq. (22):

22 $$\:{Q}_{fan}=\frac{{{\dot{\mathrm{m}}}}_{a}}{{\rho\:}_{air}}\:\:{\Delta\:}\mathrm{P}\:$$

#### Exergy analysis

The general exergy balance equation for SAH is expressed as follows^33,34^:

23 $$\:E{X}_{in}-E{X}_{out}=E{X}_{dest}$$

The exergy input ($\:E{X}_{in}$) is given by^36^:

24 $$\:E{X}_{in}=I{A}_{SAH}\left[1+\frac{1}{3}\:{\left(\frac{{T}_{amb}}{{T}_{s}}\right)}^{4}-\left.\frac{4{T}_{amb}}{3{T}_{s}}\right]\right.\:\:\:\:\:\:$$

The useful exergy gain from the SAH is calculated as follows:

25 $$\:E{X}_{out}={\dot{\mathrm{m}}}_{a}\times\:{C}_{p,air}\times\:\left[\left({T}_{a,o}-{T}_{a,i}\right)-{T}_{amb}\:ln\left.\frac{{T}_{a,o}}{{T}_{a,i}}\right]-\left(\frac{{T}_{a,o}}{{T}_{a,i}}\right){Q}_{p}\:\:\right.\:$$

The term $\:\left(\frac{{T}_{a,o}}{{T}_{a,i}}\right){Q}_{p}$ is the exergy destruction due to the pressure drop.

Based on Eqs. (24) and (25), the exergy efficiency of SAH is expressed as^3,31,37^:

26 $$\:{\eta\:}_{II}=\frac{E{x}_{out}}{E{x}_{in}}\:$$

#### Economic assessment

The total initial fixed cost was determined by accounting for both the SAH unit and the solar PV power system. The cost of each SAH unit, which included the SAH holder, wooden frame, absorber plate, glass cover, and thermal insulation (glass wool), was estimated at $60 per configuration. The solar PV system was employed as a shared power source to operate the blower during the experimental evaluation of the three SAH configurations. The PV system consists of PV panels, a charge controller, an inverter, a battery, and a blower (detailed in Table 2). The total cost of the solar PV system was $565, which was distributed equally among the three models to ensure a fair economic comparison, amounting to $188.33 per model. Therefore, the total initial cost (IC) for each model was calculated at $248.33. The procedures of calculations can be described as follows^21,38,39^:

The Capital Recovery Factor (CRF) is used to spread the initial cost over the system’s lifetime and is calculated by Hassan et al.^38^:

27 $$\:CRF=\frac{ir{\left(1+ir\right)}^{n}}{{\left(1+ir\right)}^{n}-1}$$

where i*r* is the annual interest rate (assumed to be 10%)^38^ and *n* is the number of useful years (assumed to be 20 years).

The fixed annual cost (FAC) is determined as:

28 $$\:FAC=CRF*IC$$

The system sinking fund factor (SFF) is calculated as:

29 $$\:SSF=\frac{ir}{{\left(1+ir\right)}^{n}-1}$$

The annual salvage value (ASV) is given by Hegde et al.^33^:

30 $$\:ASV=SSF*SV$$

where,

31 $$\:SV=0.2*IC$$

The annual maintenance cost (AMC) is assumed to be 10% of the FAC^40^.

The total annual cost (TAC) is computed as:

32 $$\:TAC=FAC+AMC-ASV$$

The cost of thermal unit (CTU, $/kW_th_) is calculated by Hegde et al.^33^:

33 $$\:CTU=\frac{TAC}{n*{N}_{op}*{Q}_{u,\:\:daily}}\:$$

where, $\:{N}_{op}$ is the number of operating days per year (assumed 320 days in Egypt)^34^, and $\:{Q}_{u,\:\:daily}$ is the useful heat gain per day (daily working hours were set at 6 h).

#### Enviro-economic evaluation

The average CO_2_ emission from power generation using coal is approximately 2.08 kg CO_2_/kWh. Based on this, the total annual CO_2_ mitigation ($\:{\varphi\:}_{C{O}_{2}}$) achieved by using a SAH can be estimated as follows^33^:

34 $$\:{\varphi\:}_{C{O}_{2}}=\frac{{N}_{op}*{Q}_{u,\:\:daily}*2.08}{1000}$$

where, $\:{Q}_{u,\:overall}$ is the total annual useful energy output (in kWh/year).

The enviro-economic benefit (CO_2_ mitigation price per annum, in $/year) is calculated by Hegde et al.^33^:

35 $$\:{Z}_{C{O}_{2}}={CP}_{C{O}_{2}}*{\varphi\:}_{C{O}_{2}}$$

$$\:{CP}_{C{O}_{2}}$$ is the global carbon price, assumed to be $50/ton as recommended by the World Bank to support climate action and limit global warming to 2 °C by 2030^34^.

### Uncertainty analysis

The uncertainties associated with the variables considered in the experimental analysis are summarized in Table 3. For directly measured quantities, the uncertainty values were determined based on the accuracy specifications provided by the measuring instruments. In contrast, the uncertainties of calculated parameters were evaluated using the following equation^41^.

36 $$\:{W}_{R}=\:{\left[\sum\:_{i=1}^{n}\left(\frac{\partial\:R}{\partial\:{X}_{i}}{W}_{{X}_{i}}\right)\right]}^{0.5}$$

where $\:{W}_{R}$​ represents the uncertainty associated with the calculated parameter *R*, $\:{W}_{{X}_{i}}$​​ denotes the uncertainty of the independent variable $\:{X}_{i}$​, and *n* is the number of variables influencing the result.

**Table 3:** Uncertainty of measured and calculated parameters used in the experimental analysis.

Variable	Uncertainty
Ambient temperature	± 1 °C
Solar radiation	± 5%
Wind velocity	± 5%
Outlet air temperature	± 1 °C
Mass flow rate	± 5%
Thermal efficiency	± 2.65–3.96%

## Results and discussions

**Fig. 6:**
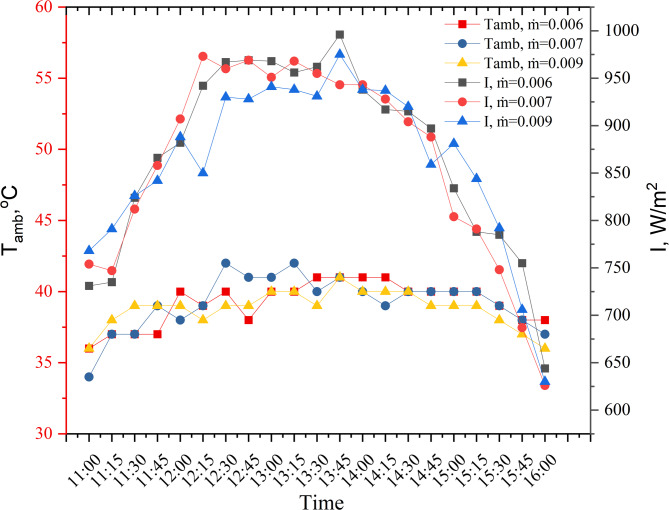
Solar radiation and ambient temperature for SAH during the experiment period in July 2025.

Figure 6 illustrates the variation in solar irradiation and ambient temperature during testing of the SAH under different mass flow rates in July 2025. Both ambient temperature and solar irradiance increased from 11:00 AM to 1:30 PM and gradually decreased from 1:30 PM to 4:00 PM. On the first test day, using a mass flow rate of 0.006 kg/s, the maximum solar irradiance reached 870 W/m^2^, and the peak ambient temperature was 39.2 °C. On the second day, with a mass flow rate of 0.007 kg/s, the maximum solar irradiance was 862.7 W/m^2^, and the ambient temperature peaked at 39.3 °C. On the third day, using a mass flow rate of 0.009 kg/s, the solar irradiance reached a maximum of 862.6 W/m^2^, with a corresponding peak ambient temperature of 38.8 °C. These observations confirm that the experiments were conducted under comparable and consistent environmental conditions across all test days.

### Compare the performance of different SAH at different mass flow rate

**Fig. 7:**
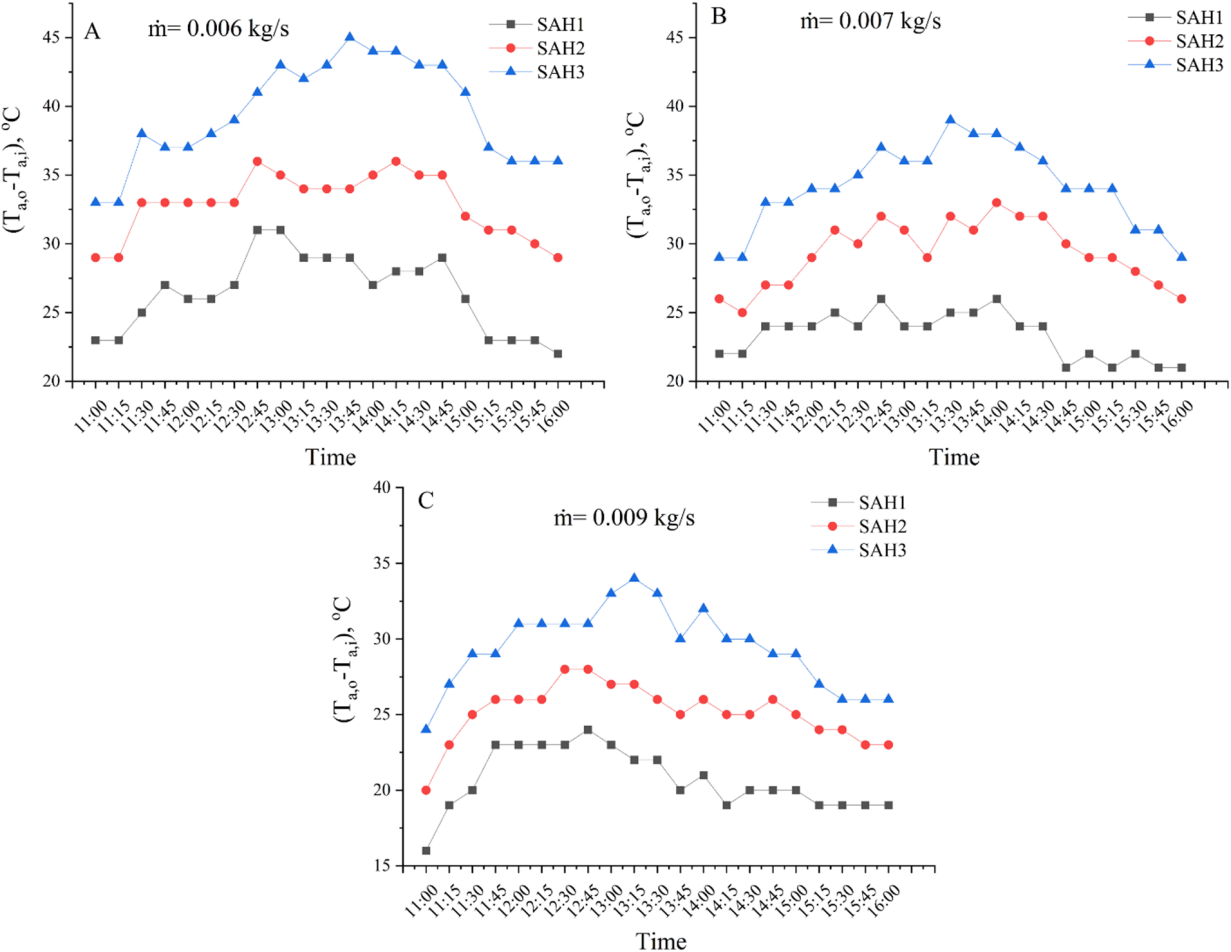
Difference in temperature between inlet and outlet for SAH1, SAH2, and SAH3 at different mass flow rates.

This section presents a comparative analysis of the energy and exergetic performance of three SAH configurations, SAH1, SAH2, and SAH3, under varying mass flow rates (0.006, 0.007, and 0.009 kg/s). The performance is evaluated based on energy output, energy efficiency, exergy output, and exergy efficiency. Figure 7 shows the temperature difference (T_a, o_−T_a, i_) for SAH1, SAH2, and SAH3 under three mass flow rates: 0.006, 0.007, and 0.009 kg/s. At a mass flow rate of 0.006 kg/s (Fig. 7A), SAH3 demonstrated the highest average temperature rise, reaching a peak of approximately 45 °C around 13:45, with a daily average of 39.5 °C. SAH2 exhibited stable and moderate performance, maintaining a temperature difference between 29 and 36 °C throughout the day, with an average of approximately 32.9 °C. SAH1 showed the lowest temperature gain, peaking near 31 °C and averaging around 26.4 °C. When the mass flow rate was increased to 0.006 kg/s and 0.009 kg/s, a general reduction in temperature difference was observed across all systems, attributed to reduced residence time of air within the SAH. The average temperature differences under 0.007 kg/s were 34.1 °C for SAH3, 29.3 °C for SAH2, and 23.4 °C for SAH1 (Fig. 7B). While the average temperature differences under 0.009 kg/s were 29.4 °C for SAH3, 25.1 °C for SAH2, and 20.7 °C for SAH1 (Fig. 7C). However, temperature difference (T_a, o_−T_a, i_) decreases with increasing mass flow rate, consistent with faster air movement reducing heat absorption time.

SAH3 consistently outperformed the other designs at both flow rates, indicating superior thermal collection and transfer characteristics. The superior performance of SAH3 is primarily attributed to the integration of V-shaped perforated fins, which function as effective turbulators within the air channel. These fins disrupt the development of the thermal boundary layer and promote the generation of longitudinal vortices. This secondary flow enhances the mixing between the heated absorber surface and the core airflow, thereby significantly increasing the convective heat transfer coefficient compared to the flat-plate designs of SAH1 and SAH2. In contrast, SAH1 was more sensitive to changes in operating conditions and consistently showed the lowest thermal performance.

**Fig. 8:**
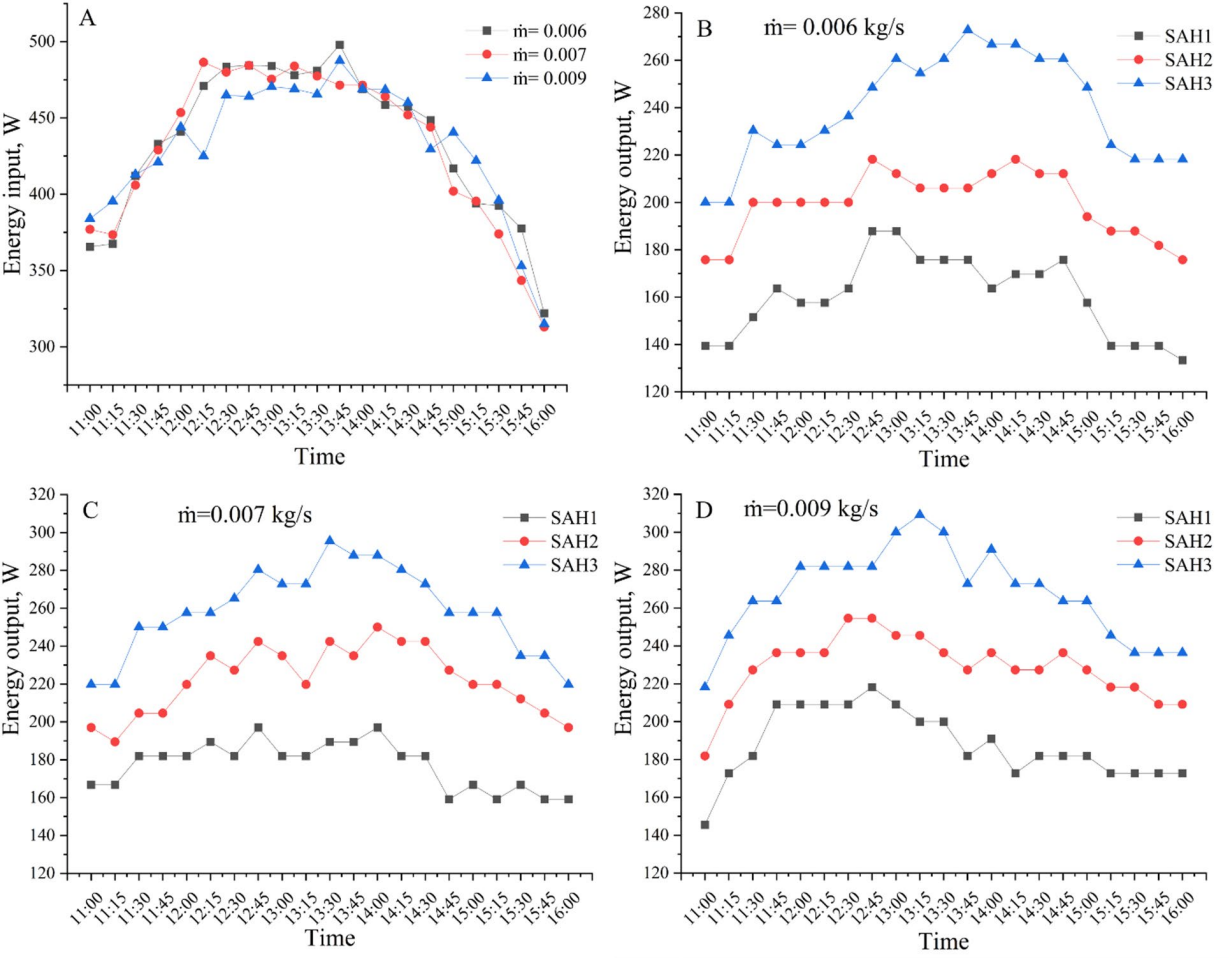
Energy input and energy output for SAH1, SAH2, and SAH3 at different mass flow rates.

**Fig. 9:**
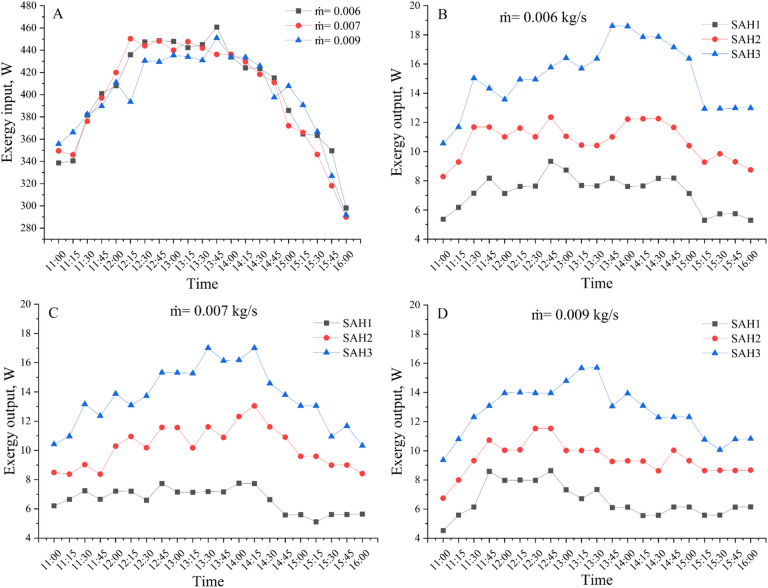
Exergy input and output exergy for SAH1, SAH2, and SAH3 at different mass flow rates.

The energy input, exergy input, output energy, and output exergy trends for the three SAH configurations (SAH1, SAH2, and SAH3) were evaluated under three mass flow rates: 0.006, 0.007, and 0.009 kg/s and plotted in Figs. 8 and 9. At all airflow rates, the energy input, exergy input, output energy, and output exergy follow a typical solar pattern, with values rising during the morning hours, peaking around solar noon (12:30–13:30), and declining in the late afternoon. The energy input and exergy input, primarily driven by incident solar radiation, remained relatively consistent among all study periods under identical environmental conditions. As shown in Fig. 8A, the average energy inputs are 435, 431.3, and 431.2 for 0.006, 0.007, and 0.009 kg/s, respectively. On the other hand, the average exergy inputs are 402.7, 399.2, and 399.3 for 0.006, 0.007, and 0.009 kg/s, respectively (Fig. 9A). The close agreement between the energy and exergy input values across all operating conditions indicates that the experiments were conducted under nearly identical environmental conditions, ensuring a fair comparison of system performance.

However, significant differences were observed in energy output and exergy output. At a mass flow rate of 0.006 kg/s (Fig. 8B), SAH3 consistently achieved the highest energy output throughout the day, peaking at approximately 272.8 W, followed by SAH2 with a peak near 218.2 W, and SAH1 with the lowest peak around 187.9 W. The maximum energy outputs under 0.007 kg/s were 296 W for SAH3, 250 W for SAH2, and 197 W for SAH1 (Fig. 8C). While the maximum energy outputs under 0.009 kg/s were 309 W for SAH3, 255 W for SAH2, and 218 W for SAH1 (Fig. 8D). As the mass flow rate increases, energy output rises in all SAH configurations due to the increased mass of air heated. On the other hand, the average exergy outputs under 0.006 kg/s were 15.1 W for SAH3, 10.7 W for SAH2, and 7.2 W for SAH1 (Fig. 9B). At a mass flow rate of 0.007 kg/s (Fig. 9C), SAH3 consistently achieved the highest exergy output throughout the day, averaging at approximately 13.7 W, followed by SAH2 with an average of 10.2 W, and SAH1 with an average of 6.6 W. While the average exergy outputs under 0.009 kg/s were 12.7 W for SAH3, 9.4 W for SAH2, and 6.5 W for SAH1 (Fig. 9D). The trend indicates that while all SAHs receive similar solar input, their ability to convert this input into useful thermal energy output and exergy output varies significantly. SAH3’s superior output suggests more effective heat transfer mechanisms and reduced thermal losses. In contrast, SAH1 exhibited a sharper decline in output during the afternoon hours, reflecting higher inefficiencies and potential heat dissipation. SAH2 maintained moderate and more stable energy output and exergy output levels, positioning it as a balanced design. Overall, the energy output and exergy output trends confirm the enhanced thermal conversion capability of SAH3, independent of fluctuations in solar input.

**Fig. 10:**
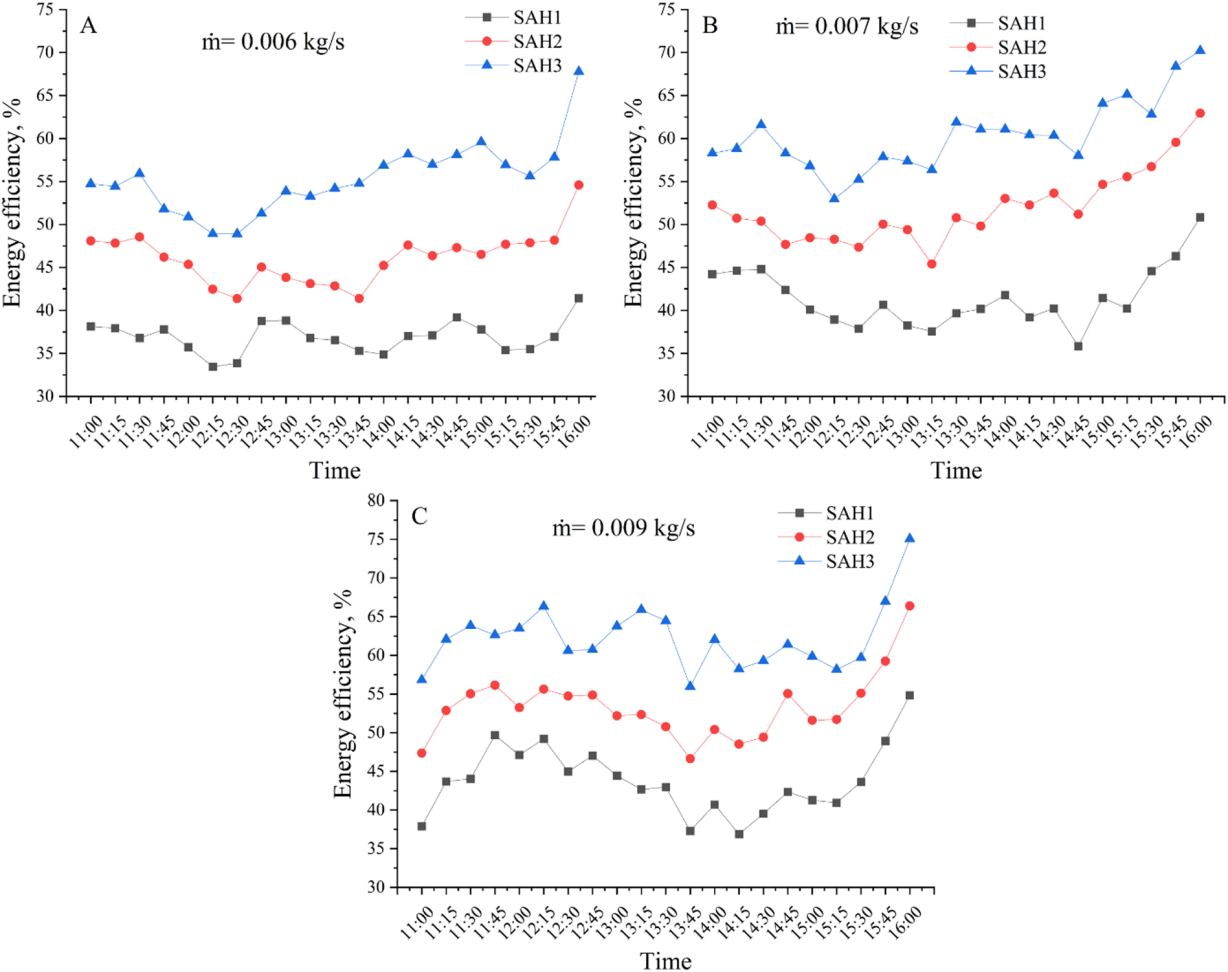
Energy efficiency for SAH1, SAH2, and SAH3 at different mass flow rates.

The energy efficiency of SAH1, SAH2, and SAH3 was analyzed across different mass flow rates (0.006, 0.007, and 0.009 kg/s) to evaluate the impact of airflow on thermal conversion performance. A consistent trend was observed in all three configurations: energy efficiency increased with the mass flow rate (Fig. 10). This is attributed to the greater mass of air being heated and carrying away more thermal energy, despite a reduction in the temperature difference due to shorter residence time. This trend highlights a fundamental trade-off in SAHs: increasing the mass flow rate shortens the air residence time and lowers the temperature difference (ΔT), but it simultaneously raises the Reynolds number and enhances flow turbulence. The resulting improvement in convective heat transfer and heat removal factor enables the system to extract and deliver a greater amount of useful thermal energy, leading to the observed increase in overall energy efficiency. At a mass flow rate of 0.006 kg/s (Fig. 10A), SAH3 consistently achieved the highest energy efficiency throughout the day, averaging approximately 0.55, followed by SAH2 with an average of 0.46, and SAH1 with the lowest energy efficiency with an average of 0.37. The average energy efficiencies at 0.007 kg/s were 0.60 for SAH3, 0.52 for SAH2, and 0.41 for SAH1 (Fig. 10B). While the average energy efficiencies at 0.009 kg/s were 0.62 for SAH3, 0.53 for SAH2, and 0.44 for SAH1 (Fig. 10C). Among the configurations, SAH3 exhibited the highest energy efficiency at all tested flow rates, with a maximum efficiency exceeding 75% at the highest mass flow rate. SAH2 demonstrated moderate efficiency, increasing steadily with mass flow rate but remaining lower than SAH3. SAH1 consistently had the lowest energy efficiency across all conditions, reflecting its limited capacity to transfer absorbed heat effectively. The trend confirms that higher flow rates enhance the thermal performance of SAHs, but the degree of improvement depends heavily on system design. SAH3’s superior efficiency trend underlines its optimized geometry and heat transfer mechanisms, making it the most effective configuration regardless of airflow rate.

**Fig. 11:**
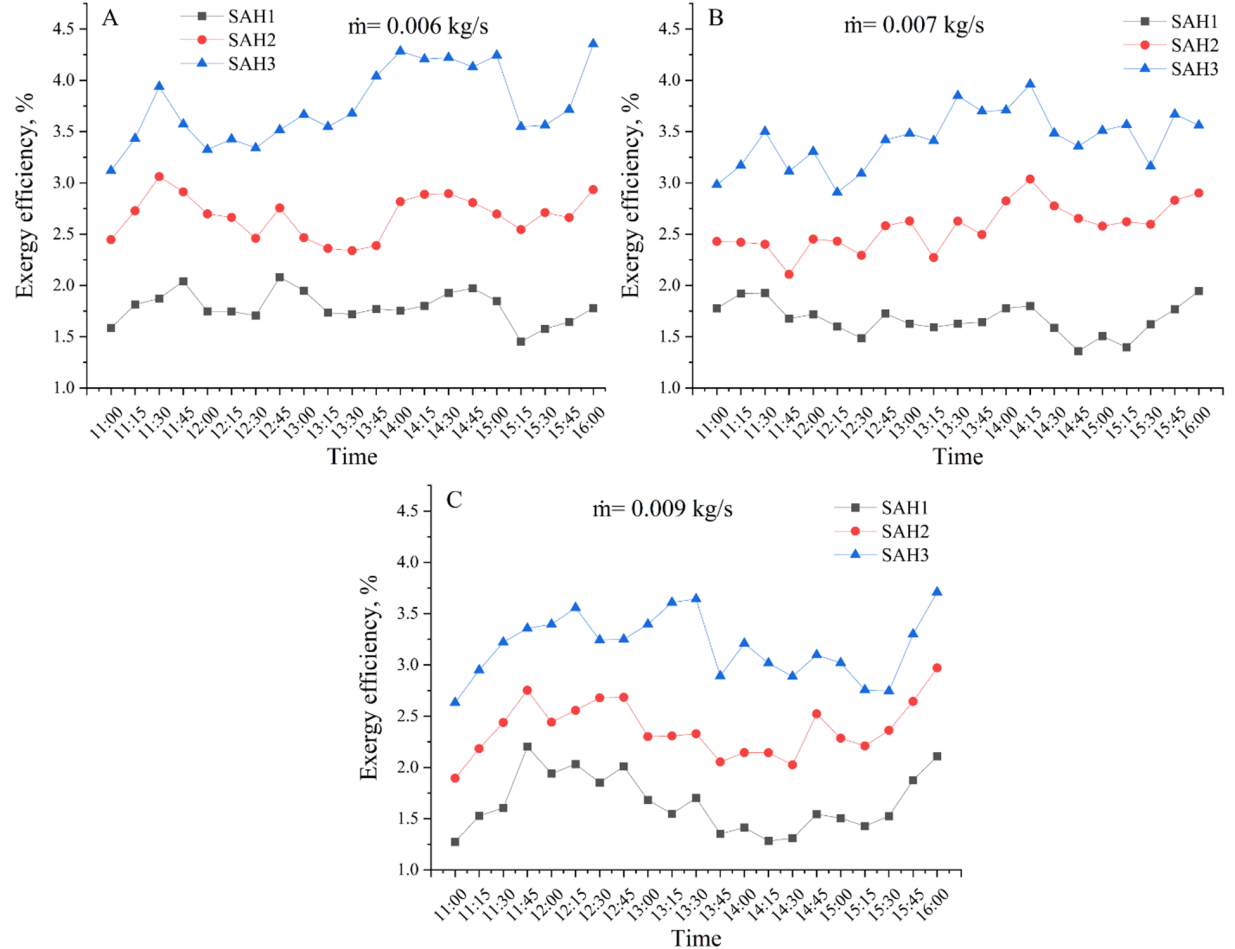
Exergy efficiency for SAH1, SAH2, and SAH3 at different mass flow rates.

The exergy efficiency of SAH1, SAH2, and SAH3 was evaluated under mass flow rates of 0.006, 0.007, and 0.009 kg/s and plotted in Fig. 11. At a mass flow rate of 0.006 kg/s (Fig. 11A), SAH3 achieved an average exergy efficiency of 3.7, followed by SAH2 with an average of 2.6, and SAH1 with the lowest exergy efficiency with an average of 1.8. The average exergy efficiencies at 0.007 kg/s were 3.4 for SAH3, 2.6 for SAH2, and 1.7 for SAH1 (Fig. 11B). While the average exergy efficiencies at 0.009 kg/s were 3.2 for SAH3, 2.4 for SAH2, and 1.6 for SAH1 (Fig. 11C). Unlike energy output or energy efficiency, which generally increase with rising mass flow rates, the exergy efficiency showed minimal variation across the tested flow conditions. For all three SAH configurations, the exergy efficiency remained relatively stable, with only slight fluctuations. SAH3 consistently demonstrated the highest exergy efficiency, followed by SAH2, while SAH1 recorded the lowest values. The lack of significant change in exergy efficiency with increasing mass flow rate can be attributed to a trade-off between higher exergy output and reduced temperature gradient at the outlet. As the mass flow rate increases, although more air is heated, the outlet temperature tends to decrease slightly due to reduced residence time, which in turn diminishes the specific exergy gained per unit of mass. These opposing effects offset each other, resulting in relatively constant exergy efficiency. This trend highlights that system design and thermal configuration play a more dominant role in determining exergy efficiency than flow rate alone, emphasizing the superior performance of SAH3 under all operating conditions.

The significant disparity between energy efficiency (reaching 62%) and exergy efficiency (averaging 3.7%) for SAH3 highlights the substantial exergy destruction inherent in the solar-to-thermal conversion process. This destruction is primarily caused by the large temperature gradient between the high-temperature solar source and the relatively low-temperature absorber plate. Furthermore, while the V-shaped fins in SAH3 enhance heat transfer, they also contribute to internal exergy losses due to increased fluid friction and pressure drop, which must be balanced against the thermal gains.

### Economic and environmental analysis

Table 4 presents the results of the economic analysis. The findings reveal that the proposed SAH3 system consistently achieves the lowest energy cost across all operating conditions when compared to SAH1 and SAH2. This confirms that SAH3 not only offers superior energy efficiency, as previously demonstrated, but also delivers a more cost-effective performance. The reduced energy cost of SAH3 is attributed to its high thermal efficiency and lower annual operating cost. Specifically, the high thermal efficiency of 62% achieved by SAH3 directly minimizes the Levelized Cost of Energy (LCOE). By maximizing the useful heat gain from the same solar aperture area and capital investment, the cost per unit of energy is significantly reduced to 0.0003 $/kWh. This demonstrates that the marginal cost of fabricating and installing V-shaped fins is negligible compared to the substantial economic benefits derived from improved thermal performance. Notably, SAH1 at a mass flow rate of 0.006 kg/s records the highest energy cost (0.00051 $/kWh), while SAH3 at 0.009 kg/s achieves the lowest energy cost (0.0003 $/kWh). These results strongly suggest that SAH3 is the most economically advantageous option among the tested configurations.

An enviro-economic analysis was conducted to evaluate the CO_2_ reduction benefits of all tested SAHs, as summarized in Table 4. The results show that the amount of mitigated CO_2_ is directly proportional to the annual energy output of each system. Among the configurations, the proposed SAH3 consistently achieved the highest CO_2_ mitigation across all operating conditions, outperforming both SAH1 and SAH2. Notably, SAH3 at a mass flow rate of 0.009 kg/s mitigated approximately 1.07 tons of CO_2_ per year, resulting in an annual carbon credit of 53.43 USD based on a carbon price of 50 USD/ton. Beyond the direct reduction in greenhouse gas emissions, the associated carbon credit value provides a tangible financial incentive that further enhances the system’s feasibility. This dual benefit underscores the role of optimized SAHs in achieving both environmental sustainability and economic profitability in decentralized heating applications.

**Table 4:** Enviro-economic results for all SAHs.

SAH type	Airflow rate (kg/s)	$$\:{Q}_{th,\:daily\:acc}$$ (kWh/day)		$$\:CTU$$ ($/kW_th_)	$$\:{Q}_{u,\:overall}$$ (kWh/year)		$$\:{\varphi\:}_{C{O}_{2}}$$ (ton Co_2_/year)	$$\:{Z}_{C{O}_{2}}$$ ($/year)
SAH1	$\dot{\mathrm{m}}$= 0.006	0.96	0.0051		307.59	0.64		31.99
	$\dot{\mathrm{m}}$= 0.007	1.06	0.0046		340.15	0.71		35.38
	$\dot{\mathrm{m}}$= 0.009	1.13	0.0043		360.80	0.75		37.52
SAH2	$\dot{\mathrm{m}}$= 0.006	1.20	0.0041		382.41	0.80		39.77
	$\dot{\mathrm{m}}$= 0.007	1.33	0.0037		426.75	0.89		44.38
	$\dot{\mathrm{m}}$= 0.009	1.37	0.0036		438.94	0.91		45.65
SAH3	$\dot{\mathrm{m}}$= 0.006	1.44	0.0034		459.45	0.96		47.78
	$\dot{\mathrm{m}}$= 0.007	1.55	0.0031		496.72	1.03		51.66
	$\dot{\mathrm{m}}$= 0.009	1.61	0.0030		513.76	1.07		53.43

### Comparative performance evaluation with previous studies

A comparative assessment of the present work with previously published SAH studies is summarized in Table 5. As shown, earlier investigations primarily focused on improving thermal efficiency through double-pass configurations, corrugated absorbers, or enhancements using porous and wire-mesh materials. For instance, Hassan et al.^42^ reported a high thermal efficiency of 88.4% for a double-pass tubular SAH; however, economic and environmental indicators were not provided, limiting the assessment of overall system sustainability. Abdelrahman et al.^34^ achieved a thermal efficiency of 53% using recyclable soda cans, accompanied by moderate exergy efficiency and CO_2_ savings, but at a higher heat cost compared with the present study. Studies incorporating corrugated or V-shaped fins, such as those by Alrashidi et al.^43^, Rajendran et al.^44^, and El-Said et al.^45^, demonstrated moderate thermal and exergy efficiencies, yet lacked a comprehensive economic or environmental evaluation. In contrast, the proposed SAH3 demonstrates a balanced and superior 4E performance. Although some reported systems achieved higher thermal efficiencies, SAH3 attains a competitive thermal efficiency of 62% while delivering a markedly lower heat cost of 0.0003 $/kWh, which is significantly lower than values reported in previous studies. Moreover, SAH3 achieves a CO_2_ mitigation potential of approximately 1.07 tons per year, confirming its environmental advantage. This comparison clearly indicates that the proposed configuration not only enhances heat transfer performance but also offers improved economic viability and environmental sustainability, distinguishing it from existing SAH designs. The SAH3 design offers a superior cost-to-benefit ratio due to its structural simplicity and low manufacturing requirements. This balance of high efficiency, low cost, and significant environmental impact confirms its suitability for large-scale adoption in sustainable thermal energy systems.

**Table 5:** Comparative 4E performance of the present SAHs with published studies.

Study	SAH configuration	Mass flow rate (kg/s)	Thermal efficiency (%)	Exergy efficiency (%)	Heat cost ($/kWh)	CO_2_ Savings (ton/year)
Hassan et al.^42^	Double-pass nabla tubular SAH	0.072	88.4	3.89%	N/A	N/A
Abdelrahman et al.^34^	Recyclable soda cans SAH	0.012	53	40.2	0.0042	0.158
Alrashidi et al.^43^	Flat plate SAH with V-shaped fins	0.011	30.9	15.1	N/A	N/A
El-Said et al.^45^	Double-pass SAH with corrugated absorber plate	0.03	34.3	1.1	0.00467	N/A
Hassan et al.^38^	CP_SAH	–	71.85%	0.975%	0.0324	3.328
Fattoum et al.^46^	SAH with a porous wire mesh	0.045	76%	12%	0.0105	1.37
Rajendran et al.^44^	SAH using V baffles	0.0384	61	N/A	N/A	N/A
Present study (SAH3)	Air chamber beneath the absorber plate integrated with perforated V-shaped fins	0.009 kg/s	62	3.7	0.0003	1.07

## Conclusion

This study experimentally investigated the performance of three SAH configurations under varying mass flow rates, focusing on 4E aspects. The tested configurations included SAH1 (airflow above the absorber plate), SAH2 (airflow beneath the absorber plate), and SAH3 (airflow beneath the absorber plate integrated with perforated V-shaped fins). Key findings from the analysis are as follows:

- SAH3 consistently exhibited higher thermal efficiency across all mass flow rates. At 0.009 kg/s, SAH3 achieved the highest average thermal efficiency of 62%, compared to 53% for SAH2 and 44% for SAH1.
- SAH3 recorded the highest average exergy of 3.7% at 0.006 kg/s, followed by SAH2 at 2.6% and SAH1 at 1.8%.
- SAH3 demonstrated the lowest energy cost, reaching 0.0003 $/kWh at 0.009 kg/s, indicating strong economic feasibility.
- SAH3 achieved the greatest CO_2_ mitigation, reducing emissions by approximately 1.07 tons per year and generating an annual carbon credit of 53.43 USD.

Overall, the proposed SAH significantly enhanced heat transfer, leading to improved energy and exergy performance while also reducing environmental and operational costs. These findings establish SAH3 as the most efficient and sustainable design among the configurations studied, particularly under higher mass flow rate conditions. This study has several limitations, including that the experiments were conducted under specific climatic conditions and within a limited range of airflow rates. Therefore, the reported performance trends may vary depending on weather conditions or seasonal variations. Furthermore, the analysis focused on steady-state operation, while transient effects resulting from fluctuations in solar radiation and ambient temperature were not considered. The long-term durability of the absorber and fin surfaces, as well as potential degradation due to dust accumulation or corrosion, were also not addressed in this work. Future studies should investigate the performance of these systems under diverse climatic conditions and for extended operating periods. Further improvements could be achieved by incorporating phase-change materials to enhance thermal storage, optimizing fin geometry and airflow distribution, and integrating the system with hybrid or photovoltaic-supported configurations.

## Data Availability

The availability of data and materials based on personal request.

## Abbreviations

$$I$$: Solar radiation (W/m^2^)
L: Air gap thickness above the absorber plate (m)
*h*: Heat transfer coefficient (W/m^2^ K)
*T*: Temperature (°C)
V: Wind velocity (m/s)
k: Thermal conductivity (W/m K)
*d*_*f*_: Gap between absorber and baseplate (m)
Nu: Nusselt number
R_a_: Rayleigh number
$$g$$: Acceleration due to gravity (m/s^2^)
$${R}_{e}$$: Reynolds number
h: Channel height
$${D}_{h}$$: Hydraulic diameter
w: Channel width
b: The mean thickness of the insulation
*Q*_*u*_: Useful heat gain (W)
SAH: Solar air heater
*A*: Area (m^2^)
$$\dot{\mathrm{m}}$$: Air mass flow rate (kg/s)
$${C}_{p}$$: Specific heat capacity (J/kg K)
*Q*_*fan*_: Fan power consumption (W)
*CF*: Conversion factor
*P*: Pressure drop across the collector (Pa)
$$E{X}_{in}$$: Total input exergy from solar radiation
$$E{X}_{out}$$: Output exergy of the heated air (W)
EX_dest_: Exergy destruction (W)
IC: Initial cost of every SAH ($)
CRF: Capital recovery factor
FAC: Fixed annual cost
SFF: Sinking fund factor
ASV: Annual salvage value
AMC: Annual maintenance cost
$${N}_{op}$$: Number of operating days per year
$$CTU$$: Cost of thermal unit ($/kW_th_)
TAC: Total annual cost
$${Q}_{u, daily}$$: Useful heat gain per day
$${\phi }_{C{O}_{2}}$$: Total annual CO_2_ mitigation

## Subscripts

n: SAH lifespan
i*r*: Annual interest rate
amb: Ambient
$$g$$: Glass cover
$$ab$$: Absorber plate
*con*: Conduction
*r*: Radiation
$$a$$: Air
$$bp$$: Baseplate
$$c$$: Convection
$$s$$: Sun
ins: Insulation
i: *Inlet*
o: *Outlet*
eff: Effective

## Greek

$$\alpha$$: Absorptivity
$${\eta }_{th}$$: *Thermal efficiency*
$${\eta }_{II}$$: Exergy efficiency
$$\tau$$: Transmittance
$$\varepsilon$$: Emissivity
θ: Angle of inclination
ρ: Density (kg/m^3^)
β: Thermal-expansion coefficient (1/K)
μ: Dynamic Viscosity of air (kg/m s)
